# Radiomics-guided multimodal MRI fusion framework for non-enhanced glioblastoma noninvasive identification

**DOI:** 10.1371/journal.pdig.0001662

**Published:** 2026-08-21

**Authors:** Shuai Liu, Xuewei Mao, Xing Liu, Shaowu Li, Shiwen Weng, Chenxing Wu, Wei Shan, Zhen Wu

**Affiliations:** 1 Department of Radiation Oncology, Beijing Tiantan Hospital, Capital Medical University, Beijing, China; 2 Beijing Neurosurgical Institute, Capital Medical University, Beijing, China; 3 School of Automation and the Shandong Key Laboratory of Industrial Control Technology, Qingdao University, Qingdao, China; 4 Department of Neuroradiology, Beijing Tiantan Hospital, Capital Medical University, Beijing, China; 5 Department of Neurology, Beijing Tiantan Hospital, Capital Medical University, Beijing, China; 6 Department of Neurosurgery, Sanbo Brain Hospital, Capital Medical University, Beijing, China; 7 Department of Neurosurgery, Beijing Tiantan Hospital, Capital Medical University, Beijing, China; Tianjin Medical University General Hospital, CHINA

## Abstract

This study aimed to develop and externally validate a radiomics-based machine learning framework for the noninvasive differentiation of non-enhanced glioblastoma (NE-GBM) from astrocytoma, IDH-mutant, grade 2 (IDHm-A2), thereby addressing the diagnostic limitations of qualitative MRI and the challenge of data scarcity. This diagnostic study used a radiomics-based machine learning framework, with integrated histopathological and molecular diagnosis serving as the reference standard. We retrospectively enrolled 84 glioma patients (July 2021-November 2022) from our center, comprising 34 pathologically confirmed NE‑GBM. Radiomic features were extracted from T1, T2, FLAIR, and ADC sequences. The framework was developed to differentiate NE‑GBM from IDHm‑A2 and was subsequently validated on an internal test cohort (n = 22) and an external test cohort (n = 12). Diagnostic performance was assessed using ROC analysis and decision curves, and model predictions were interpreted via SHapley Additive exPlanations (SHAP). A total of 118 patients (47 NE‑GBM, 71 IDHm‑A2) were evaluated. Fifty‑four radiomic features and age were selected for model construction. The optimized model demonstrated strong discriminatory performance, with the internal test cohort achieving an AUC of 0.935 and an accuracy of 91.0%, and the external test cohort yielding an AUC of 0.969 and an accuracy of 90.0%. The proposed framework provides an accurate, noninvasive tool for distinguishing NE‑GBM from IDHm‑A2, directly addressing this critical diagnostic challenge.

## Author summary

Recent advances in molecular biology have led to the recognition of molecular glioblastoma, a subtype that frequently manifests as non-enhancing glioblastoma (NE-GBM) on MRI. This phenotype makes it difficult to distinguish from IDH-mutant grade 2 astrocytoma (IDHm-A2) using conventional imaging. Here, we developed a radiomics-based machine learning framework that analyzes multiparametric MRI (T1, T2, FLAIR, ADC) to provide a noninvasive differential diagnosis. Given the rarity of this disease entity, a total of 118 patients were enrolled from two neuro-oncology centers. The main analytical workflow comprised radiomic feature extraction, feature selection using LASSO regression, and the construction and tuning of multiple classifiers. The model achieved consistently high area under the curve (AUC) values in both internal cross-validation and two independent external test cohorts. SHAP analysis further identified key contributing radiomic features, thereby enhancing model interpretability. This approach addresses a critical gap in the preoperative assessment of non-enhancing gliomas and demonstrates the clinical value of quantitative imaging biomarkers for molecular phenotyping in neuro-oncology.

## Introduction

Glioblastoma has long attracted significant attention as one of the most lethal primary malignant brain tumors [1]. Advances in molecular biology have substantially deepened our understanding of gliomas, revealing that different molecular subtypes exhibit distinct microstructural characteristics and that certain radiological features are associated with specific molecular profiles [2–4]. This convergence of histological heterogeneity and imaging phenotype opens the possibility of leveraging imaging data as a noninvasive, reproducible tool for molecular subtyping.

**Fig 1:**
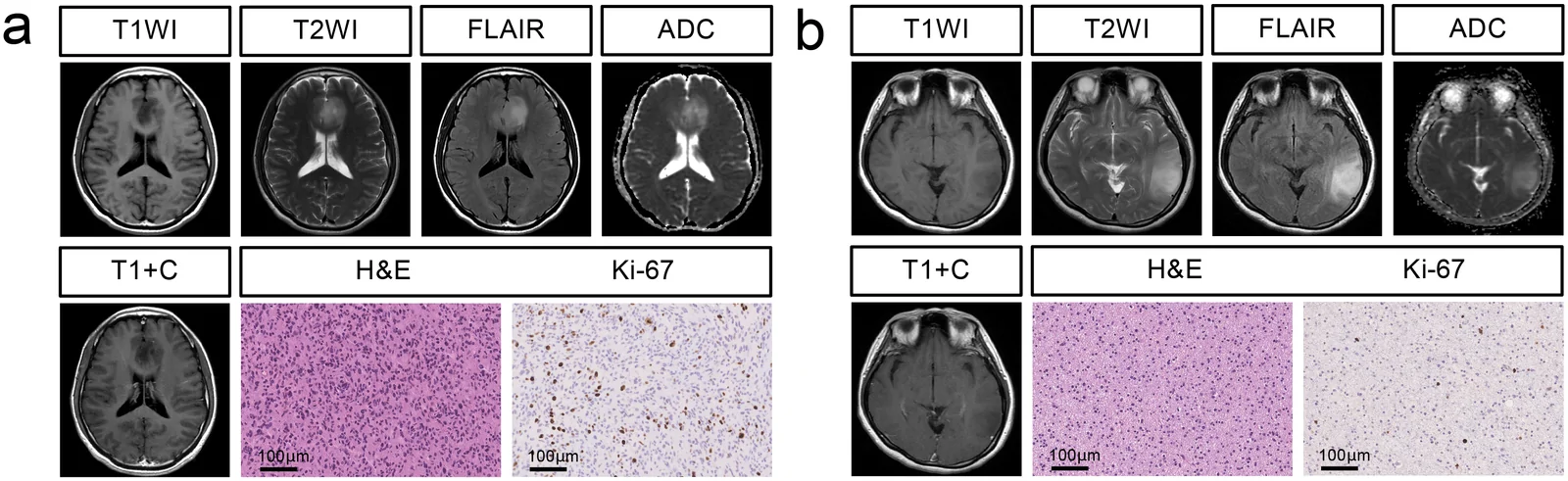
Multimodality MRI and pathological findings of representative cases. **(a)** Non‑enhanced glioblastoma (NE‑GBM). **(b)** Astrocytoma, IDH-mutant, grade 2 (IDHm-A2). H&E, hematoxylin‑eosin staining.

The fifth edition of the WHO Classification of Tumors of the Central Nervous System (WHO CNS5) recognizes a subgroup of molecularly diagnosed glioblastoma that paradoxically displays histological features resembling lower-grade gliomas (WHO grades 2 and 3) [5]. This subtype, termed non‑enhanced glioblastoma (NE‑GBM), frequently presents as a lesion without contrast enhancement on presurgical MRI (Fig 1a), mimicking astrocytoma, IDH-mutant, grade 2 (IDHm-A2, Fig 1b). Diagnostic difficulty is further compounded by the localized, mild edema of NE-GBM (Fig 1), whereas its conventional counterpart typically shows extensive peritumoral edema. Consequently, NE-GBM is often misdiagnosed as IDHm-A2 on qualitative MRI assessment. Given that patients with IDHm-A2 have a significantly better prognosis than those with glioblastoma [1,6], accurate preoperative differentiation is essential. This enables balancing the extent of resection with preservation of neurological function, thereby guiding individualized surgical decision-making and potentially improving patient outcomes.

Given the limitations of conventional qualitative MRI in differentiating glioma molecular subtypes and the challenges of interpreting non-enhancing diffuse lesions, radiomics-based frameworks offer a paradigm-shifting solution. Through high-throughput extraction of quantitative imaging features, machine learning (ML) captures subtle pathological variations undetectable by conventional radiological assessment, enabling systematic tumor characterization beyond human visual capabilities. Previous studies have demonstrated the good performance of radiomic models in predicting molecular subtypes of various tumors, including lung cancer [7], breast cancer [8], colorectal cancer [9], renal cell carcinoma [10] and medulloblastoma [11]. For glioma, Kickingereder et al. [12] established ML classification models based on MR imaging features to predict key molecular characteristics of glioblastoma. Using a radiomic model, Lu et al. determined the histology and molecular subtypes of gliomas with good performance [13].

Despite significant advances in artificial intelligence, radiomics and oncology, current literature remains disproportionately focused on glioblastoma grading and molecular subtyping, with insufficient attention to the critical diagnostic distinction between NE-GBM and IDHm-A2. This knowledge gap poses challenges in optimizing preoperative clinical decision-making for ambiguous neuroimaging presentations. To address this unmet need, we propose a novel methodology integrating systematic radiomic analysis of multimodal MRI datasets with clinical parameter fusion for noninvasive NE-GBM identification. Our framework employs radiomic feature extraction to quantify tumor heterogeneity across T1-weighted, T2-weighted, fluid-attenuated inversion recovery (FLAIR), and apparent diffusion coefficient (ADC) imaging, followed by an ensemble ML architecture optimized for discriminating NE-GBM from IDHm-A2. The model’s biological interpretability is reinforced by SHapley Additive exPlanations (SHAP), which identify the key radiomic-clinical determinants, while its generalizability is rigorously assessed through external validation across multi-institutional cohorts. This paradigm advances precision neuroimaging diagnostics and provides a practical clinical tool for preoperative risk stratification, enabling personalized surgical planning and adjuvant therapy optimization that may improve survival outcomes.

## Method

### Study participants and ethics statement

The Ethics Committees of the Beijing Tiantan Hospital and the Sanbo Brain Hospital approved this study, and written consent was obtained from all enrolled patients.

In this study, we analyzed 84 patients from the Beijing Tiantan Hospital for model training, including 34 patients with NE-GBM and 50 with IDHm-A2, who were treated between July 2021 and November 2022. Two independent test sets were utilized to evaluate the diagnostic performance and reproducibility of the predictive model: the Tiantan test cohort enrolled 22 patients from the Beijing Tiantan Hospital who were treated between January 2020 and June 2021, and the Sanbo test cohort enrolled 12 patients from the Sanbo Brain Hospital who were treated between July 2020 and December 2022. All enrolled adult patients met the following criteria: (1) newly diagnosed IDH-wildtype glioblastoma and IDHm-A2 according to the WHO CNS5, with no history of craniotomy or biopsy; (2) presurgical T1-, T2-, and contrast-enhanced T1-weighted imaging, FLAIR, and ADC imaging; and (3) lesions showing no enhancement on MRI. Among the 121 eligible patients, two from the Beijing Tiantan Hospital and one from the Sanbo Brain Hospital were excluded due to poor image quality.

### Detection of molecular status

Targeted sequencing, including somatic mutations and copy number variations, was performed in all of the patients for model training, and the methods are described in detail in a previous publication [14]. In the two independent test sets, except for the targeted sequencing mentioned above, other methods, including pyrosequencing [15], immunochemistry, and fluorescence in situ hybridization, were used. Details of the methods are shown in S1 Table.

### MRI protocol and radiomic feature extraction

The details of the MRI protocol are presented in S2 Table. The tumor regions of interest (ROIs), defined as regions showing abnormal signal on FLAIR, were manually delineated by a neuro-oncologist using 3D slicer software [16]. Next, all ROIs were examined by a senior neuro-radiologist (SWL) with 30 years of experience, who was blinded to the clinical information of the patients. The radiomic features of the ROIs for each patient were extracted using Pyradiomics (version 3.0.1) [17]. Before feature extraction, the images were resampled to a voxel size of 1 mm^3^ and discretized to a bin width of 0.1. Then features were calculated on the images after the wavelet and Laplacian of Gaussian filters, which resulted in 1,239 radiomic features per imaging sequence. The radiomic features of three basic groups included 16 shapes and 19 first-order and 75 second-order features, which were subdivided by the underlying grey-level matrices: grey-level co-occurrence matrix, grey-level dependence matrix, grey-level run-length matrix, grey-level size zone matrix, and neighbouring grey-tone difference matrix.

### Machine learning approach

**Fig 2:**
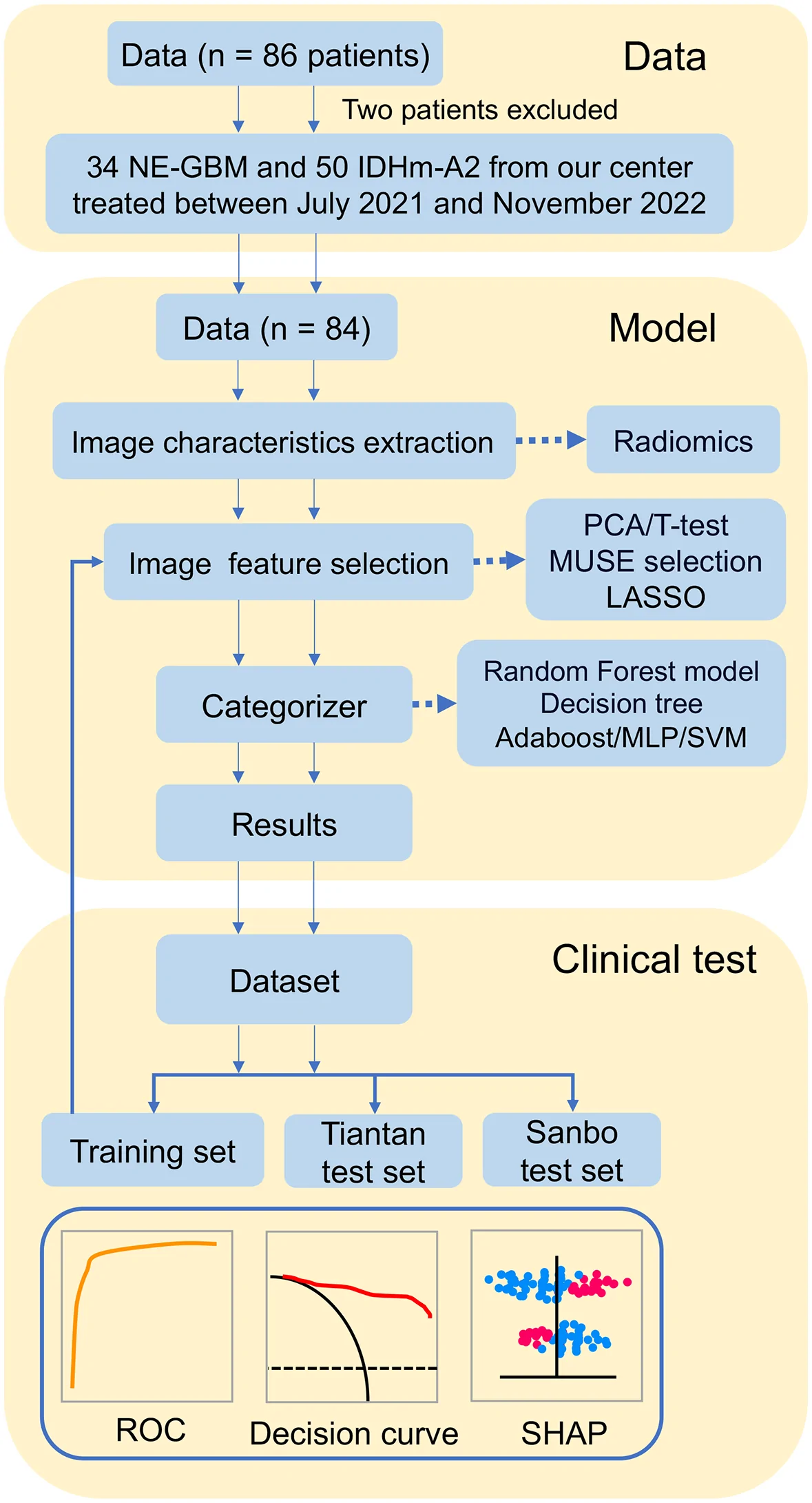
Schematic representation of cohort stratification and methodological pipeline.

A flowchart of the ML approach is shown in Fig 2. Data pre-processing and model training were performed using the Scikit-learn library module (version 1.0.2). First, all radiomic features were min–max normalized, scaling the feature values to the [0, 1] range to eliminate dimensional differences between features and enhance the model’s training effectiveness. Next, feature selection was carried out to reduce the number of highly correlated features. We conducted a systematic comparative analysis of various feature selection methodologies, including principal component analysis, T-Test, minimum uncertainty and sample elimination based binary feature selection, and the least absolute shrinkage and selection operator (LASSO), to identify the optimal feature subset that maximizes predictive accuracy while mitigating overfitting in the ML pipeline.

ML models were constructed using multiple classifiers, including random forest, decision trees, AdaBoost, multilayer perceptron, and support vector machine (SVM) with five-fold cross-validation. Given that all cases were pathologically confirmed, the model’s diagnostic accuracy was rigorously validated against the ground truth. Model parameter tuning was performed using GridSearchCV until the average validation performance did not further improve and was then applied to the independent test datasets. The model performance was evaluated using the area under the receiver operating characteristic (ROC) curve (AUC). The model interpretation was performed using SHAP [18].

### Tumor lesion overlay maps

The FLAIR images and the corresponding ROIs of the training cohort were registered to the Montreal Neurological Institute template using SPM12 (https://www.fil.ion.ucl.ac.uk/spm/software/spm12/). All of the registered ROIs were examined by the senior neuroradiologist. Tumor lesion overlay maps were generated using MRIcroGL (https://www.nitrc.org/projects/mricrogl/).

### Statistical analysis

Statistical analyses and figure plotting were performed using Python. The chi-square test or Fisher’s exact test was used to determine significant differences in binary variables. The Wilcoxon test was used to compare continuous variables. Differences with *p*-values < 0.05 were considered statistically significant.

## Result

### Patient characteristics

In the model training dataset, NE-GBM was found to be more likely to occur in older patients than IDHm-A2 (median 53 vs. 35, *p* < 0.001), which was also observed in the Tiantan test cohort (median 50 vs. 34, *p* = 0.005). Meanwhile, no significant difference in the sex ratio was observed between the NE-GBM and IDHm-A2 groups. The volume of NE-GBM was significantly larger than that of IDHm-A2 (*p* = 0.015); however, no significant differences were observed between the Tiantan and Sanbo test cohorts. The overlay maps describe the locations of the NE-GBM and IDHm-A2, both of which mostly involved the left frontal, insular, and temporal lobes (S1 Fig). Details of the clinical information of all patients enrolled in the current study are shown in Table 1.

**Table 1:** Clinical characteristics of enrolled patients at each institution.

Characteristic	Training cohort			Tiantan test cohort			Sanbo test cohort		
	NE-GBM	IDHm-A2	*P*-value	NE-GBM	IDHm-A2	*P*-value	NE-GBM	IDHm-A2	*P*-value
Number	34	50		8	14		5	7	
Age			**<0.001**			**0.005**			0.530
Median (range)	53 (18-72)	35 (22-65)		50 (23-58)	34 (19-47)		49 (41-61)	42 (29-69)	
Sex			0.662			0.649			0.081
Female (%)	14 (41)	23 (46)		2 (25)	6 (43)		5 (100)	3 (43)	
Male (%)	20 (59)	27 (54)		6 (75)	8 (57)		0	4 (57)	
Volume, cm^3^			**0.015**			0.095			0.432
Mean±SD	35.29 ± 33.80	19.38 ± 21.27		13.52 ± 8.17	35.38 ± 27.07		54.98 ± 39.25	41.73 ± 50.61	

### Feature selection for modeling

A total of 4956 radiomic features (extracted from four MRI sequences) and two clinical features (patient age and sex) were included in the feature selection process. We compared feature selection methods with the different classifiers, and the LASSO regression method that achieved the best performance (Table 2) with 98.89% redundant feature reduction was selected for further model construction. Using this method, we screened 55 most relevant features corresponding to nonzero coefficient, which included 10 features from T1 images, 15 from T2 images, 14 from FLAIR images, 15 from ADC images, and one clinical feature (age) (S3 Table).

**Table 2:** Comparison of different feature selection methods with multiple classifiers in the cross-validation test set of training cohort.

Methods	Random forest	Decision trees	Adaboost	MLP	SVM
None	0.885	0.662	0.844	0.855	0.863
PCA	0.619	0.673	0.581	0.547	0.494
T-Test	0.891	0.722	0.846	0.915	0.887
MUSE	0.815	0.718	0.811	0.830	0.814
LASSO	0.956	0.723	0.936	0.999	0.998

Abbreviation: PCA, principal component analysis; MUSE, minimum uncertainty and sample elimination based binary feature selection; LASSO, least absolute shrinkage and selection operator; MLP, multi-layer perceptron; SVM, support vector machine.

### Predictive performance of the model

**Fig 3:**
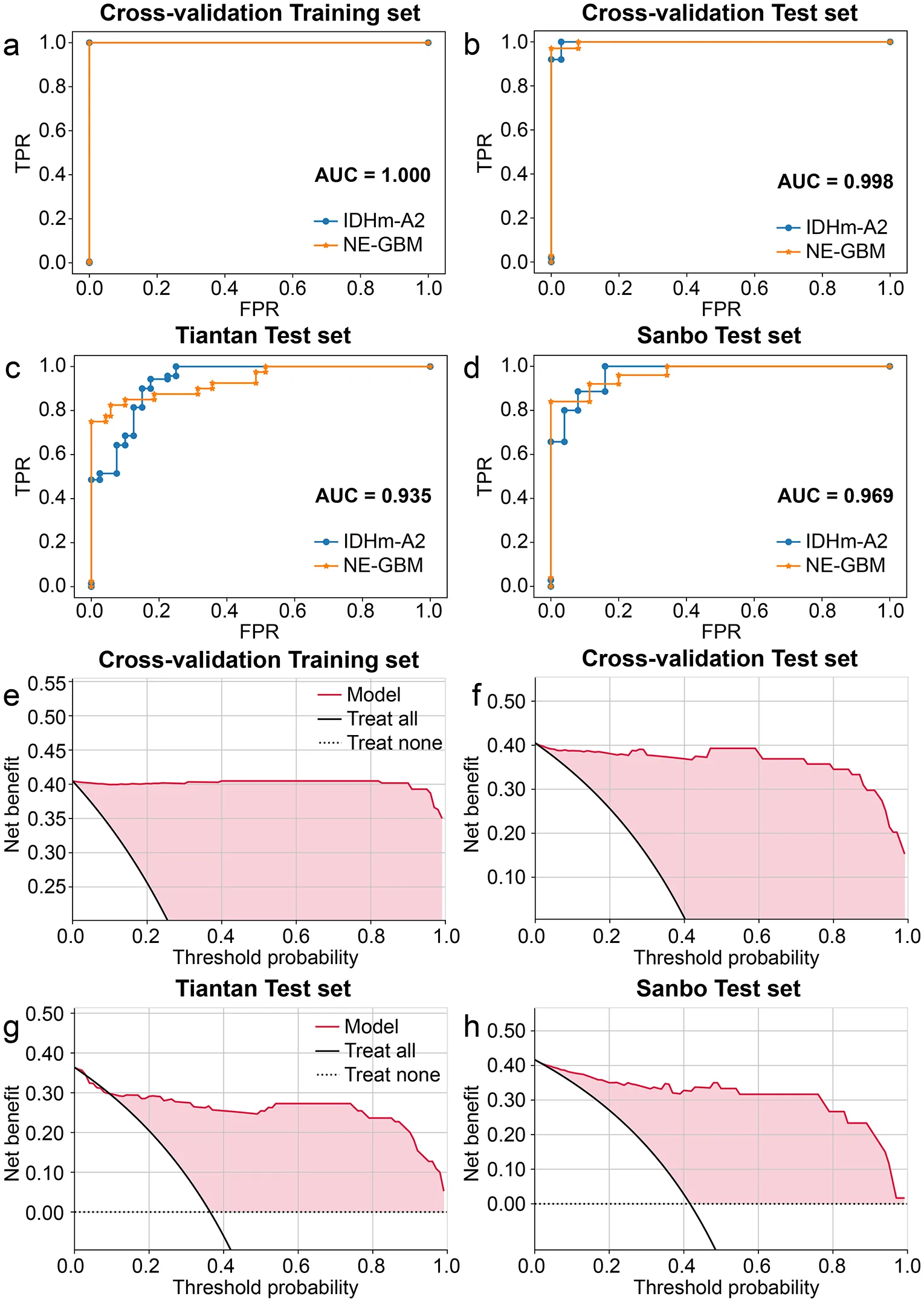
ROC curves and decision curves of the predictive model. **(a–d)** ROC curves for the cross‑validation training set **(a)**, cross‑validation test set **(b)**, Tiantan test set **(c)**, and Sanbo test set **(d)**. **(e–h)** Decision curves for the cross‑validation training set **(e)**, cross‑validation test set **(f)**, Tiantan test set **(g)**, and Sanbo test set **(h)**.

The diagnostic performance of the five classifiers trained on their respective optimal hyperparameters in the training and test cohorts is listed in Tables 3 and 4, respectively. As shown, the multilayer perceptron and SVM classifiers achieved superior results compared to the other classifiers. The final ensemble model, which integrates the predictions of the five classifiers, achieved an AUC of 1.000 (Fig 3a) and an accuracy of 1.000 in the cross‑validation training set; in the cross‑validation test set, it achieved an AUC of 0.998 (Fig 3b) and an accuracy of 0.980; in the Tiantan test set, an AUC of 0.935 (Fig 3c) and an accuracy of 0.910; and in the Sanbo test set, an AUC of 0.969 (Fig 3d) and an accuracy of 0.900. The decision curves were further analyzed (Fig 3e-h).

**Table 3:** Diagnostic performance of the 5 classifiers in the training cohort.

Classifiers	Cross-validation Training set				Cross-validation Test set			
	Accuracy	Macro avg F1	Weighted avg F1	AUC	Accuracy	Macro avg F1	Weighted avg F1	AUC
Random forest	0.980	0.980	0.980	0.998	0.890	0.890	0.890	0.956
Decision trees	0.940	0.940	0.940	0.938	0.730	0.720	0.730	0.723
Adaboost	0.970	0.970	0.970	0.998	0.870	0.860	0.870	0.936
MLP	1.000	1.000	1.000	1.000	0.980	0.980	0.980	0.999
SVM	1.000	1.000	1.000	1.000	0.980	0.980	0.980	0.998

Abbreviation: MLP, multilayer perceptron; SVM, support vector machine; Macro avg F1, macro average of F1 score; Weighted avg F1, weighted average of F1 score.

**Table 4:** Diagnostic performance of the 5 classifiers in the test cohorts.

Classifiers	Tiantan Test set				Sanbo Test set			
	Accuracy	Macro avg F1	Weighted avg F1	AUC	Accuracy	Macro avg F1	Weighted avg F1	AUC
Random forest	0.900	0.880	0.900	0.853	0.800	0.790	0.800	0.900
Decision trees	0.650	0.630	0.650	0.627	0.670	0.640	0.660	0.640
Adaboost	0.860	0.840	0.860	0.836	0.850	0.850	0.850	0.929
MLP	0.910	0.900	0.910	0.854	0.870	0.860	0.870	0.939
SVM	0.910	0.900	0.910	0.933	0.900	0.890	0.900	0.974

### Model interpretation by SHAP

**Fig 4:**
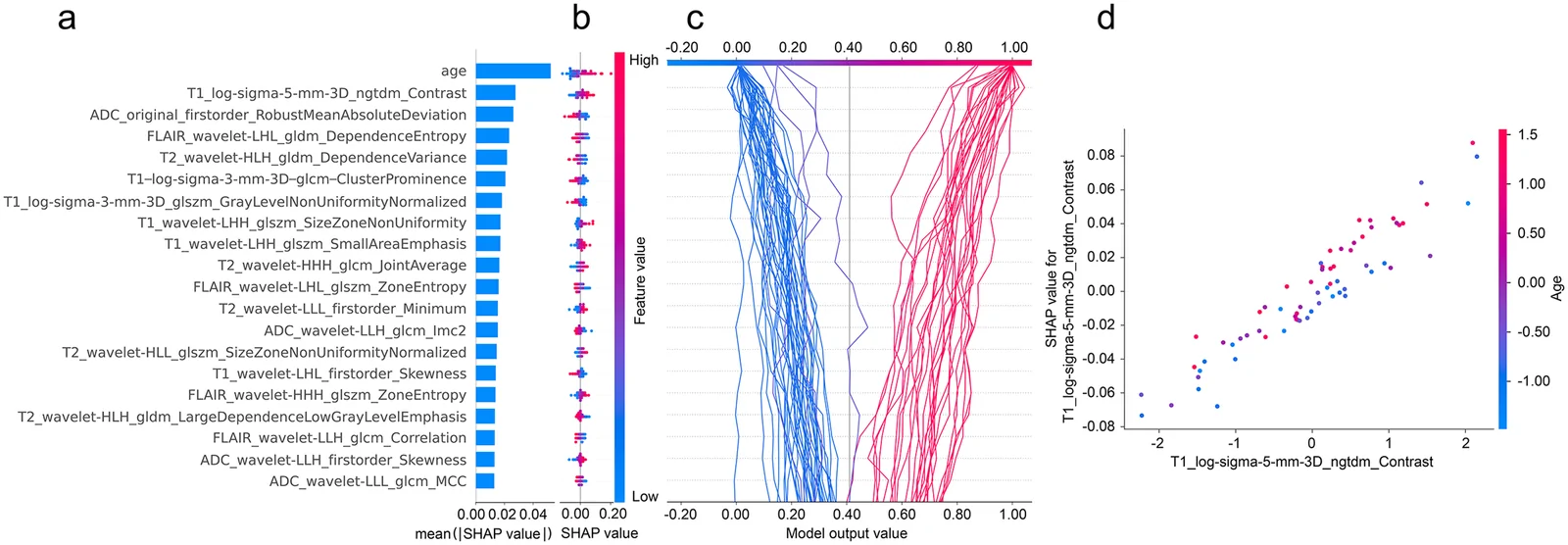
Model interpretation by SHapley Additive exPlanations (SHAP). **(a)** Bar plot: the global importance of each feature, which is shown as the mean absolute value for that feature over all the included cases; **(b)** Beeswarm plot: summary of how the 20 top features impact the model’s output; **(c)** Decision plot: display the process of how model making a prediction; **(d)** Scatter plot: describing the interaction effect between the top radiomic feature (T1_log-sigma-5-mm-3D_ngtdm_Contrast) and age.

The SHAP values of the top 20 features for the model building were calculated and are shown as a bar plot (Fig 4a), a beeswarm plot (Fig 4b), and a decision plot (Fig 4c). We further explored the interaction between the top two features using a scatter plot (Fig 4d).

**Fig 5:**
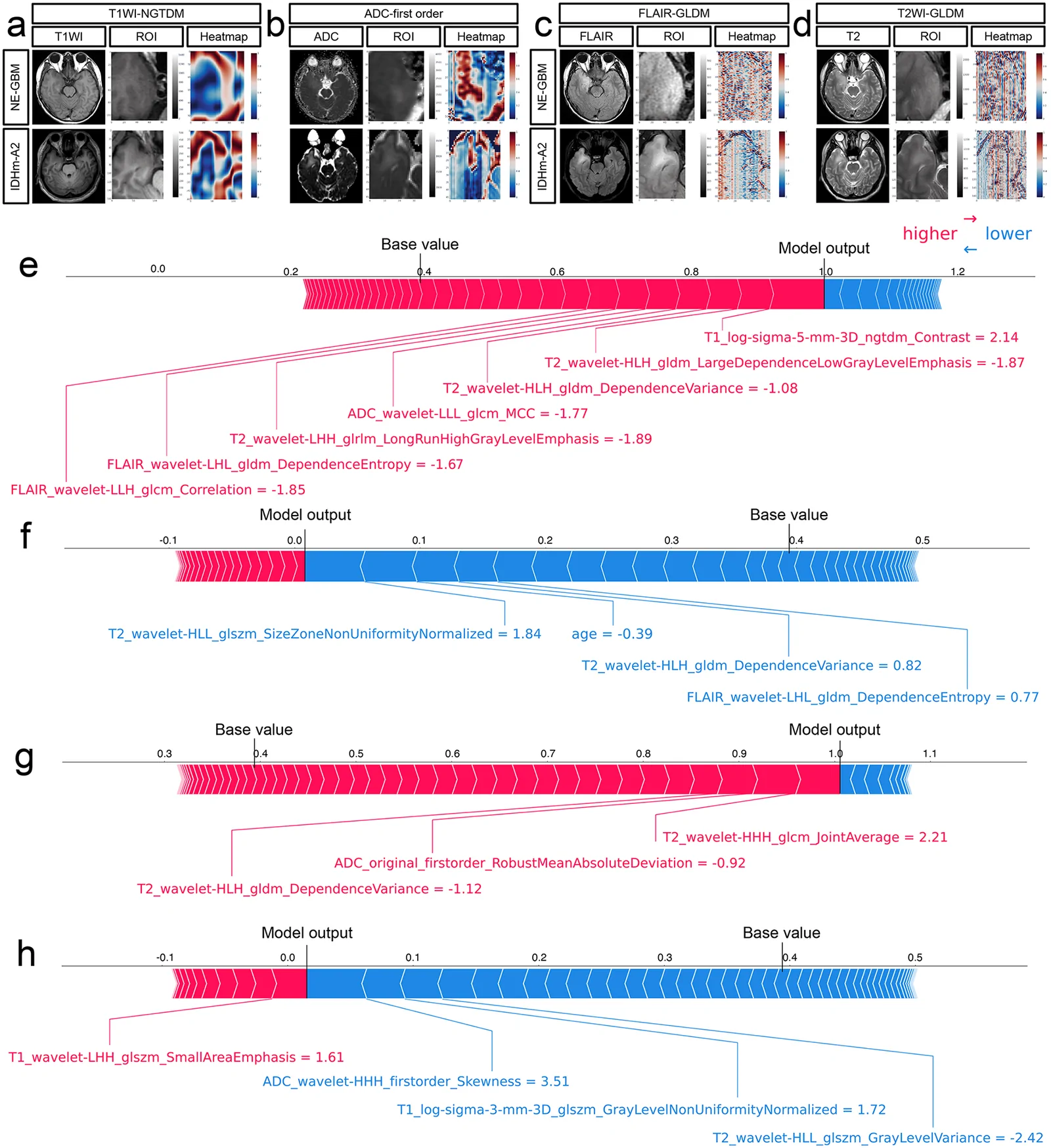
Heatmaps of the top four radiomic features of a patient with NE-GBM (upper row) and a patient with IDHm-A2 (lower row). T1_log-sigma-5-mm-3D_ngtdm_Contrast (T1-NGTDM) **(a)**, ADC_original_firstorder_RobustMeanAbsoluteDeviation (ADC-first order) **(b)**, FLAIR_wavelet-LHL_gldm_DependenceEntropy (FLAIR-GLDM) **(c)**, and T2_wavelet-HLH_gldm_DependenceVariance (T2-GLDM) **(d)**. The force plots of the above two patients are shown as (e) and **(f)**, respectively. (g) and (h) are the force plots from the patient with NE-GBM in Fig 1(a) and the patient with IDHm-A2 in Fig 1(b), respectively. These plots indicate how a feature pushes the model prediction, and the colour of the arrow represents the contribution of the feature as positive (red) or negative (blue).

To further visualize the modeling process, we present two cases with heat maps of the top four radiomic features (Fig 5a-d). The force plots indicate that the radiomic features were capable of differentiating NE-GBM from IDHm-A2, which shows a similar MRI appearance. Fig 5g and 5h show the force plots from the information in Fig 1a and 1b, respectively.

## Discussion

In this study, we developed and externally validated a radiomics-based ML framework for the noninvasive differentiation of NE-GBM from IDHm-A2. The clinical challenge of distinguishing these two entities on conventional MRI is both difficult and critical, as misdiagnosis may result in surgical interventions that are either overly aggressive, or not timely enough and inadequate in their extent of resection. Our model demonstrated consistently strong discriminatory performance in both internal and external test cohorts, highlighting its substantial clinical value.

The integration of molecular markers into diagnostic frameworks such as WHO CNS5 has enhanced diagnostic precision and profoundly influenced treatment decisions [19]. Molecularly defined glioblastoma, which can histologically resemble lower-grade glioma, exemplifies how genetic features now serve as a critical complement to morphological criteria [5]. This progress has created a pressing clinical need for noninvasive methods to predict molecular subtypes. While histopathological and molecular analyses remain the diagnostic gold standard, they require invasive tissue sampling that is not always feasible longitudinally. A noninvasive imaging surrogate that captures a tumor’s molecular profile could bridge the gap between increasingly refined molecular classification and the demands of patient management [20–22]. By demonstrating that radiomic features can distinguish between molecularly distinct entities such as NE-GBM and IDHm-A2, our study represents a step toward this broader vision of imaging-based molecular phenotyping.

Addressing this need, the present work concentrates on a diagnostically ambiguous phenotype—non‑enhancing lesions that cannot be reliably classified by conventional radiological criteria for glioblastoma. Our findings indicate that quantitative radiomic features derived from multiparametric MRI can reveal subtle textural and volumetric differences between NE-GBM and IDHm-A2 that are imperceptible on qualitative assessment. The ML model integrating radiomic and clinical features achieved promising discriminatory performance, substantially improving diagnostic accuracy and reproducibility in the challenging context of overlapping imaging appearances (Fig 1). Age was retained in the final model, consistent with the significantly older age of NE-GBM patients (median 53 vs. 35 years; *p* < 0.001, training set). Beyond age, the model’s performance was driven by 54 radiomic features from the four MRI sequences. SHAP analysis confirmed that features from T1 and ADC sequences ranked among the top contributors, underscoring the added value of multiparametric quantification.

Accurate preoperative differentiation of NE-GBM and IDHm-A2 has profound surgical implications. For tumors associated with prolonged survival, brain plasticity enables functional reorganization [23], so that preserving neurological function may be prioritized over maximizing the extent of resection. Slow-growing tumors allow time for more complete functional remapping, enabling a staged surgical strategy with an initial subtotal resection to preserve function, followed by a second operation after cortical reorganization [24]. Conversely, for tumors with a dismal prognosis such as glioblastoma, early maximal safe resection is recommended [25]. Achieving full decompression directly reduces tumor burden, relieves mass effect, and allows safer high‑dose radiotherapy to the smaller residual volume. Thus, accurate differentiation of NE-GBM from IDHm-A2 is critical for individualized surgical planning, highlighting the practical value of the noninvasive diagnostic framework developed in this study.

An additional motivation for radiomics-based diagnostics lies in the inherent limitations of tissue sampling, particularly biopsy. Such specimens represent only a minute fraction of the tumor volume and, given the marked spatial and temporal heterogeneity of gliomas [26], may fail to capture the full biological landscape. Consequently, biopsy-based molecular diagnosis has a risk of underestimating or overestimating tumor grade and prognosis [27,28]. In contrast, advanced MRI combined with radiomic analysis provides a macroscopic assessment of the entire lesion and its peritumoral environment, yielding information that better reflects overall tumor biology than a single regional sample. Our approach leverages this advantage by fusing features from multiple MRI sequences to comprehensively characterize tumor heterogeneity, offering a complementary perspective to tissue-based diagnostics. This integration of whole-lesion imaging and regional molecular analysis may ultimately enable more accurate and representative tumor characterization.

### Limitation

This study has several limitations. The low incidence of NE-GBM inherently limits the sample size, which precludes the use of data-intensive deep learning architectures and raises the risk of overfitting in high-dimensional radiomic analyses. To mitigate this, we validated the model on independent test cohorts. The robust performance observed across these external datasets suggests that overfitting was substantially controlled and that the radiomic signature retains cross‑source discriminative power. Nonetheless, the retrospective design and still limited cohort sizes warrant cautious interpretation, as the high feature-to-sample ratio remains relatively high. Addressing data scarcity will require multicenter collaboration to establish standardized, diverse glioma datasets that enable more extensive external validation [29]. In addition, manual delineation, while potentially more accurate for gliomas with diffuse boundaries, compromises reproducibility. Developing in-house automated algorithms that convert clinical expertise into a standardized segmentation workflow would substantially address this limitation. Finally, although our framework is built upon conventional techniques, it offers substantial clinical utility. It demonstrates robust performance on small datasets and enables rapid computation suitable for time-sensitive clinical workflows. Importantly, the integration of SHAP provides transparent interpretability by identifying key imaging markers, thereby facilitating clinician interpretation of MRI findings. Overall, future work should develop novel deep learning architectures that integrate systematic augmentation strategies and the harmonization of multi-center imaging data to improve diagnostic robustness while ensuring clinical feasibility [30,31].

## Conclusion

In this study, we developed a radiomics‑based ML framework that noninvasively distinguishes NE‑GBM from IDHm‑A2 on preoperative multimodal MRI. This approach may help personalize surgical decision‑making and advance imaging‑based molecular subtyping.

## Supporting information

S1 Table Methods of detecting molecular status for diagnosis. (DOCX)

S2 Table MRI acquisition parameters and protocols for T1, T2, FLAIR, and ADC sequences. (DOCX)

S3 Table List of significant radiomic features to differentiate NE-GBM from IDHm-A2. (DOCX)

S1 Fig Lesion overlay maps of NE-GBM and IDHm-A2 from the training cohort. (DOCX)
