# Supplementary material for: Radiomics-guided multimodal MRI fusion framework for non-enhanced glioblastoma noninvasive identification
Source: PLOS Digit Health. 2026 Aug 21;5(8):e0001662. doi: 10.1371/journal.pdig.0001662 (PMC13496412; doi:10.1371/journal.pdig.0001662)
Supplement: Supplementary file 1 [file pdig.0001662.s001.docx]

**S1 Table. Methods of detecting molecular status for diagnosis.**

| Methods | Genes | Tiantan test | Sanbo test |
| --- | --- | --- | --- |
| Targeted sequencing | Panel | 8 | 2 |
| Pyrosequencing | IDH1/2, TERTp, MGMT | 14 | - |
| NGS | Panel | - | 3 |
| FISH | 1p/19q | 14 | - |
|  | CDKN2A/B | 9 | 7 |
| Immunochemistry | IDH | - | 7 |
|  | ATRX | - | 7 |

Abbreviation: NGS, next-generation sequencing; FISH, fluorescence in situ hybridization.
