# Supplementary material for: Radiomics-guided multimodal MRI fusion framework for non-enhanced glioblastoma noninvasive identification
Source: PLOS Digit Health. 2026 Aug 21;5(8):e0001662. doi: 10.1371/journal.pdig.0001662 (PMC13496412; doi:10.1371/journal.pdig.0001662)
Supplement: Supplementary file 2 [file pdig.0001662.s002.docx]

**S2 Table. MRI acquisition parameters and protocols for T1, T2, FLAIR, and ADC sequences.**

| Centers | Manufacturer  (number) | Sequence | Repetition time (ms) | Echo time (ms) | Flip angle | Thickness  (mm) | Matrix |
| --- | --- | --- | --- | --- | --- | --- | --- |
| Training  cohort | GE (20) | T1 | 2275 | 28.6 | 111 | 5.5 | 320*192 |
|  |  | T2 | 7385 | 106.9 | 142 | 5.5 | 384*384 |
|  |  | FLAIR | 8000 | 146.3 | 111 | 5.5 | 256*224 |
|  |  | ADC | 2300 | 63.2 | 90 | 5.5 | 128*128 |
|  | SIEMENS (30) | T1 | 2120 | 8.6 | 145 | 5.0 | 256*198 |
|  |  | T2 | 4500 | 99.0 | 120 | 5.0 | 320*256 |
|  |  | FLAIR | 7500 | 85.0 | 150 | 5.0 | 256*186 |
|  |  | ADC | 4100 | 98.0 | 90 | 5.0 | 156*129 |
|  | Philips (34) | T1 | 6.61 | 3.0 | 8 | 5.0 | 220*218 |
|  |  | T2 | 3100 | 114.2 | 90 | 5.0 | 288*96 |
|  |  | FLAIR | 9000 | 130.0 | 90 | 5.0 | 256*191 |
|  |  | ADC | 2342 | 66.0 | 90 | 5.0 | 114*87 |
| Tiantan  test  cohort | GE (11) | T1 | 2275 | 28.6 | 111 | 5.5 | 320*192 |
|  |  | T2 | 7385 | 106.9 | 142 | 5.5 | 384*384 |
|  |  | FLAIR | 8000 | 146.3 | 111 | 5.5 | 256*224 |
|  |  | ADC | 2300 | 63.2 | 90 | 5.5 | 128*128 |
|  | SIEMENS (5) | T1 | 2120 | 8.6 | 145 | 5.0 | 256*198 |
|  |  | T2 | 4500 | 99.0 | 120 | 5.0 | 320*256 |
|  |  | FLAIR | 7500 | 85.0 | 150 | 5.0 | 256*186 |
|  |  | ADC | 4100 | 98.0 | 90 | 5.0 | 156*129 |
|  | Philips (6) | T1 | 6.61 | 3.0 | 8 | 5.0 | 220*218 |
|  |  | T2 | 3100 | 114.2 | 90 | 5.0 | 288*96 |
|  |  | FLAIR | 9000 | 130.0 | 90 | 5.0 | 256*191 |
|  |  | ADC | 2342 | 66.0 | 90 | 5.0 | 114*87 |
| Sanbo  test  cohort | GE (7) | T1 | 1736 | 25.4 | 111 | 5.0 | 352*256 |
|  |  | T2 | 6014 | 106.4 | 111 | 5.0 | 468*272 |
|  |  | FLAIR | 11000 | 139.0 | 160 | 5.0 | 320*224 |
|  |  | ADC | 6263 | 77.8 | 90 | 5.0 | 128*130 |
|  | Philips (5) | T1 | 651 | 15.0 | 69 | 5.0 | 256*229 |
|  |  | T2 | 4361 | 90.0 | 90 | 5.0 | 356*278 |
|  |  | FLAIR | 8500 | 95.0 | 90 | 5.0 | 308*246 |
|  |  | ADC | 3250 | 82.4 | 90 | 6.0 | 128*178 |
