# Supplementary material for: Radiomics-guided multimodal MRI fusion framework for non-enhanced glioblastoma noninvasive identification
Source: PLOS Digit Health. 2026 Aug 21;5(8):e0001662. doi: 10.1371/journal.pdig.0001662 (PMC13496412; doi:10.1371/journal.pdig.0001662)
Supplement: Supplementary file 3 [file pdig.0001662.s003.docx]

**S3 Table. List of significant radiomic features to differentiate NE-GBM from IDHm-A2.**

| Feature type | Image | Feature category | | Feature |
| --- | --- | --- | --- | --- |
| Clinical feature |  |  |  | Age |
| Radiomic feature | FLAIR | Log-sigma-4-mm-3D | First-order | Interquartile Range |
|  |  | Wavelet - LLH | GLCM | Correlation |
|  |  |  |  | MCC |
|  |  | HLH |  | Imc1 |
|  |  |  | GLRLM | Short Run High Gray Level Emphasis |
|  |  | LHL | GLDM | Dependence Entropy |
|  |  |  | GLSZM | Zone Entropy |
|  |  | LHH |  | Small Area Emphasis |
|  |  |  |  | Small Area Low Gray Level Emphasis |
|  |  | HLL |  | Small Area High Gray Level Emphasis |
|  |  | HHL |  | Gray Level Non-uniformity Normalized |
|  |  |  |  | Gray Level Variance |
|  |  |  |  | Small Area High Gray Level Emphasis |
|  |  | HHH |  | Zone Entropy |
|  | T1 | Log-sigma-2-mm-3D | GLSZM | Gray Level Non-uniformity Normalized |
|  |  | Log-sigma-3-mm-3D |  | Gray Level Non-uniformity Normalized |
|  |  |  | GLCM | Cluster Prominence |
|  |  | Log-sigma-5-mm-3D | NGTDM | Contrast |
|  |  | Wavelet - LHL | First-order | Skewness |
|  |  | HLH |  | Skewness |
|  |  | LHH | GLSZM | Size Zone Non-uniformity |
|  |  |  |  | Small Area Emphasis |
|  |  | LLL | GLRLM | Long Run Emphasis |
|  |  |  | NGTDM | Complexity |
|  | T2 | Log-sigma-2-mm-3D | First-order | Skewness |
|  |  | Wavelet - HHL |  | Median |
|  |  | HHH |  | Interquartile Range |
|  |  |  | GLCM | Joint Average |
|  |  |  | GLSZM | Size Zone Non-uniformity |
|  |  | LLL |  | Large Area High Gray Level Emphasis |
|  |  |  | First-order | Minimum |
|  |  | LLH |  | High Gray Level Zone Emphasis |
|  |  |  |  | Low Gray Level Zone Emphasis |
|  |  | HLL |  | Gray Level Variance |
|  |  |  |  | Size Zone Non-uniformity Normalized |
|  |  | LHH | GLRLM | Long Run High Gray Level Emphasis |
|  |  | HLH |  | Long Run Low Gray Level Emphasis |
|  |  |  | GLDM | Dependence Variance |
|  |  |  |  | Large Dependence Low Gray Level Emphasis |
|  | ADC | Original | First-order | Robust Mean Absolute Deviation |
|  |  | Log-sigma-2-mm-3D | NGTDM | Busyness |
|  |  | Log-sigma-3-mm-3D | First-order | Minimum |
|  |  | Log-sigma-4-mm-3D | NGTDM | Busyness |
|  |  | Log-sigma-5-mm-3D | First-order | Skewness |
|  |  | Wavelet - LLH |  | Skewness |
|  |  | HHH |  | Skewness |
|  |  |  | GLCM | Imc2 |
|  |  | LLL |  | MCC |
|  |  |  | GLDM | Large Dependence Low Gray Level Emphasis |
|  |  | HLL | GLSZM | Small Area Emphasis |
|  |  | HHL |  | Small Area Emphasis |
|  |  |  |  | Small Area High Gray Level Emphasis |
|  |  | HLH | GLRLM | Gray Level Non-uniformity Normalized |
|  |  |  |  | Gray Level Variance |
