# Supplementary material for: Radiomics-guided multimodal MRI fusion framework for non-enhanced glioblastoma noninvasive identification
Source: PLOS Digit Health. 2026 Aug 21;5(8):e0001662. doi: 10.1371/journal.pdig.0001662 (PMC13496412; doi:10.1371/journal.pdig.0001662)
Supplement: Supplementary file 4 [file pdig.0001662.s004.docx]

**S1 Fig. Lesion overlay maps of NE-GBM and IDHm-A2 from the training cohort (n = 84).**

The colour bars from blue to red indicate a higher number of patients involving the brain area.
